# Ribosomal/nucleolar stress induction regulates tert-Butyl hydroperoxide (tBHP) mediated oxidative stress in *Anopheles gambiae* midguts

**DOI:** 10.1186/s13104-019-4196-1

**Published:** 2019-03-29

**Authors:** Brian B. Tarimo, Bernadette A. Hritzo, Henry Chun Hin Law, Dingyin Tao, Rebecca Pastrana-Mena, Stefan M. Kanzok, Joram J. Buza, Rhoel R. Dinglasan

**Affiliations:** 1 Department of Health and Biomedical Sciences, Nelson Mandela-African Institution of Science and Technology, Tengeru, Arusha, 23302 Tanzania; 2 W. Harry Feinstone Department of Molecular Microbiology & Immunology & the Malaria Research Institute, Johns Hopkins Bloomberg School of Public Health, Baltimore, MD 21205 USA; 3 Department of Environmental Health and Ecological Sciences, Ifakara Health Institute, Dar es Salaam, 14112 Tanzania; 4 Emerging Pathogens Institute, Department of Infectious Diseases & Immunology, College of Veterinary Medicine, University of Florida, 2055 Mowry Road, Rm 375, Gainesville, FL 32611 USA; 5 Department of Biology, Loyola University Chicago, Chicago, IL 60660 USA

**Keywords:** *Anopheles gambiae*, Malaria, Nucleolar stress, Oxidative stress, Ribosomal stress, tert-Butyl hydroperoxide, Thioredoxin, Transmission-blocking, *Plasmodium*

## Abstract

**Objective:**

A fundamental understanding of redox homeostasis in *Anopheles gambiae* midgut cells under different oxidative conditions is missing. Such knowledge can aid in the development of new malaria transmission-blocking strategies aimed at disrupting natural homeostatic processes in the mosquito during *Plasmodium* parasite uptake (i.e. blood feeding). The aim of this study was to understand how the *An. gambiae* midgut regulates oxidative stress to reactive oxygen species (ROS), especially to a potent ROS-inducer such as tert-Butyl hydroperoxide (tBHP).

**Results:**

Initial studies using quantitative immunoblot indicated that the expression of the classical antioxidant protein *An. gambiae* thioredoxin-1 (*Ag*Trx-1) remained unchanged across challenges with different concentrations of tBHP suggesting that additional mechanisms to regulate ROS may be involved. We therefore conducted a global proteomic survey, which revealed that *An. gambiae* midguts under low (50 μM) and high (200 μM) tBHP concentrations were enriched in proteins indicative of ribosomal/nucleolar stress. Ribosomal stress is an inherent cellular response to an imbalance in ribosomal proteins (RPs) due to cellular stress such as oxidative stress. Our data suggest that ribosomal/nucleolar stress is the primary cellular response in *An. gambiae* midguts under tBHP challenge. Considering these results, we discuss harnessing the ribosomal stress response as a potential malaria transmission-blocking strategy.

**Electronic supplementary material:**

The online version of this article (10.1186/s13104-019-4196-1) contains supplementary material, which is available to authorized users.

## Introduction

The sporogonic life cycle of *Plasmodium* in the mosquito is primarily extracellular and therefore, the parasites are directly and constantly exposed to reactive oxygen and nitrogen species, ROS and RNS, respectively. ROS and RNS are produced in part by mosquito’s immune system in response to invasion of its midgut epithelial cells by the parasite [1–3], vertebrate immune factors present in the ingested blood [4, 5], and natural digestion of hemoglobin present in the ingested blood [6, 7]. This highly oxidative environment, results in a population bottleneck for the parasite during development in the mosquito vector [8, 9].

**Fig. 1:**
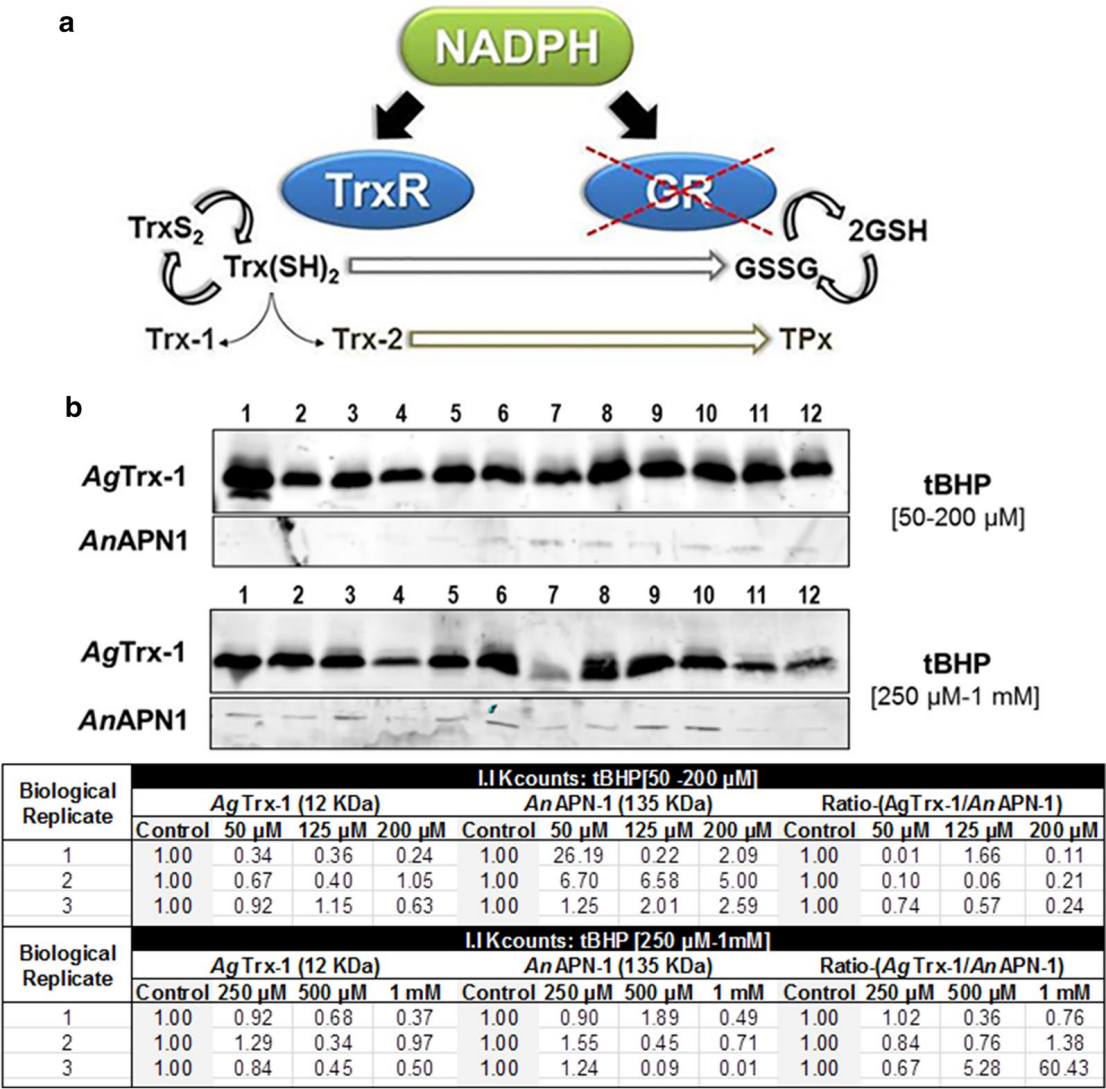
**a** Interactions between the Trx and GSH systems in redox homeostasis in *Anopheles* mosquitoes. GR is absent in the GSH system of *Anopheles* mosquitoes and is crossed out to convey this point. Therefore, *Anopheles* mosquitoes and other dipterans recycle glutathione disulfide through a dithiol-disulfide exchange with reduced thioredoxin. Reduced thioredoxin is recycled from its oxidized form by thioredoxin reductase thus maintaining sufficient levels of itself for subsequent glutathione disulfide recycling. *GSSG* glutathione disulfide, *GSH* glutathione, *GR* glutathione reductase, *NADPH* reduced nicotinamide dinucleotide phosphate, *TrxR* thioredoxin reductase, *TrxS*_*2*_ thioredoxin disulfide, *Trx(SH)*_*2*_ reduced thioredoxin, *Trx-1* thioredoxin-1, *Trx-2* thioredoxin-2, and *TPx* thioredoxin peroxidase. **b** *Ag*Trx-1 protein expression in *An. gambiae* midgut epithelial cells. Immunoblot with α-*Ag*Trx-1 antiserum of female *An. gambiae* midgut lysates obtained by incubation of midguts (5 per sample) under varied concentrations of tBHP in ex vivo organ culture media for 15 min. Female *An. gambiae* midgut lysates treated with ex vivo organ culture media (lanes 1, 5, and 9), 50 μM t-BHP (lanes 2, 6, and 10), 125 μM tBHP (lanes 3, 7, and 11), and 200 μM tBHP (lanes 4, 8, and 12) for the upper panel. Immunoblot with α-*Ag*Trx-1 antiserum of female *An. gambiae* midgut lysates obtained by incubation of midguts (5 per sample) under varied concentrations of tBHP in ex vivo organ culture media for 15 min. Female *An. gambiae* midgut lysates treated with ex vivo organ culture media (lanes 1, 5, and 9), 250 μM tBHP (lanes 2, 6, and 10), 500 μM tBHP (lanes 3, 7, and 11), and 1 mM tBHP (lane 5, 9, and 13) for the lower panel. Lanes 1–4 (biological replicate 1), lanes 5–8 (biological replicate 2), lanes 9–12 (biological replicate 3). *An*APN1 (~ 135 kDa), as a loading control is shown below each treatment column. Signal intensity was calculated in K counts mm^2^ (lower table) using LiCOR Odyssey Analytical software (Additional file 1). *P*-values (*P *≤ 0.05) were calculated by the parametric one-way analysis of variance (ANOVA) followed by Bonferroni’s correction

To maintain redox homeostasis, organisms possess the thioredoxin (Trx) and glutathione (GSH) systems as prominent mechanisms against oxidative stress. The GSH system involves the tripeptide, GSH, and in its antioxidant activity, GSH is converted to glutathione disulfide (GSSG) [10]. This oxidized form is converted back to the reduced form by the nicotinamide adenine dinucleotide phosphate-dependent flavoenzyme glutathione reductase (NADPH-GR) [11]. The Trx system is comprised of thioredoxins (Trxs), and thioredoxin reductase (TrxR) [12, 13]. Trxs are small (12 kDa) and ubiquitous thiol proteins. Trxs cycle between a disulfide and a dithiol form, catalyzed by TrxR [14]. *An. gambiae* and *An. stephensi* mosquitoes regulate Trx- and GSH-dependent antioxidants to protect midgut epithelial cells against ROS and RNS [15, 16]. Notably, *Anopheles* mosquitoes and other dipterans lack the flavoenzyme GR of the GSH pathway and utilize the Trx system to recycle GSSG to GSH (Fig. 1a) [17].

Little is known about Trx at the molecular level in *Anopheles* mosquitoes despite its importance in redox homeostasis in midgut epithelial cells under different oxidative conditions. In this report, we used an ex vivo midgut culture model to first investigate *An. gambiae* thioredoxin-1 (*Ag*Trx-1) protein expression in response to ROS challenge. Contrary to our expectations, we did not observe an upregulation in *Ag*Trx-1 across various concentrations of a ROS challenge. We then expanded our exploration to other redox homeostasis pathways by capturing the global midgut proteomic expression profile, with the aim of understanding organ-level regulation following exposure to the ROS- inducer, tert-Butyl hydroperoxide (tBHP).

## Main text

### Results

#### *Ag*Trx-1 protein expression levels

The lack of GR and utilization instead of the Trx system for GSSH recycling underscores the importance of Trx system in an antioxidant response in dipterans. As Trx-1 is one of main components of the Trx system, it therefore must play an essential role in this antioxidant response [17, 18]. We performed a quantitative immunoblot analysis of *Ag*Trx-1 protein expression in midguts that were previously exposed to the ROS producing agent tBHP. See Additional file 1 on Materials and methods for detailed explanation on ex vivo organ culture media used, mosquito rearing, experimental treatments, ROS induction assays, SDS-PAGE and immunoblot analysis.

A distinct clear band was observed at M_r_ of ~ 12 kDa across all the treatment groups and biological replicates, which corresponds to the M_r_ of *Ag*Trx-1 (Fig. 1b). Protein doublets observed in the western blot may reflect multimer of *Ag*Trx-1 or another cellular target of the antiserum used [19]. *Ag*Trx-1 protein expression level (K-counts, Fig. 1b lower panel), measured as relative expression to the loading control *Anopheline* aminopeptidase-1 (*An*APN1), did not exhibit any significant difference in *An. gambiae* midguts incubated with different concentrations of tBHP when compared to untreated controls (*P* value = 0.1695; Fig. 1b and Additional file 2). There was no significant change in the *Ag*Trx-1 expression when the tBHP concentration was increased from 250 μM to 1 mM (*P*-value = 0.4525; Fig. 1b and Additional file 2).

#### Global proteomic profiles of *An. gambiae* midguts

The absence of significant regulation in *Ag*Trx-1 expression level prompted us to expand our investigation into the antioxidant response. To this end we analyzed the global proteomic profile in midgut epithelial cells in response to varying tBHP challenges. See Additional file 1 on Materials and methods for detailed explanation on liquid chromatography–tandem mass spectrometry (LC–MS/MS) process: from protein sample preparation, separation and ionization of peptides by LC, their analysis by MS, fragmentation of selected peptides and analysis of the resulting MS/MS spectra and data analysis, including identification and quantification of proteins from several detected peptides (Additional file 3).

**Fig. 2:**
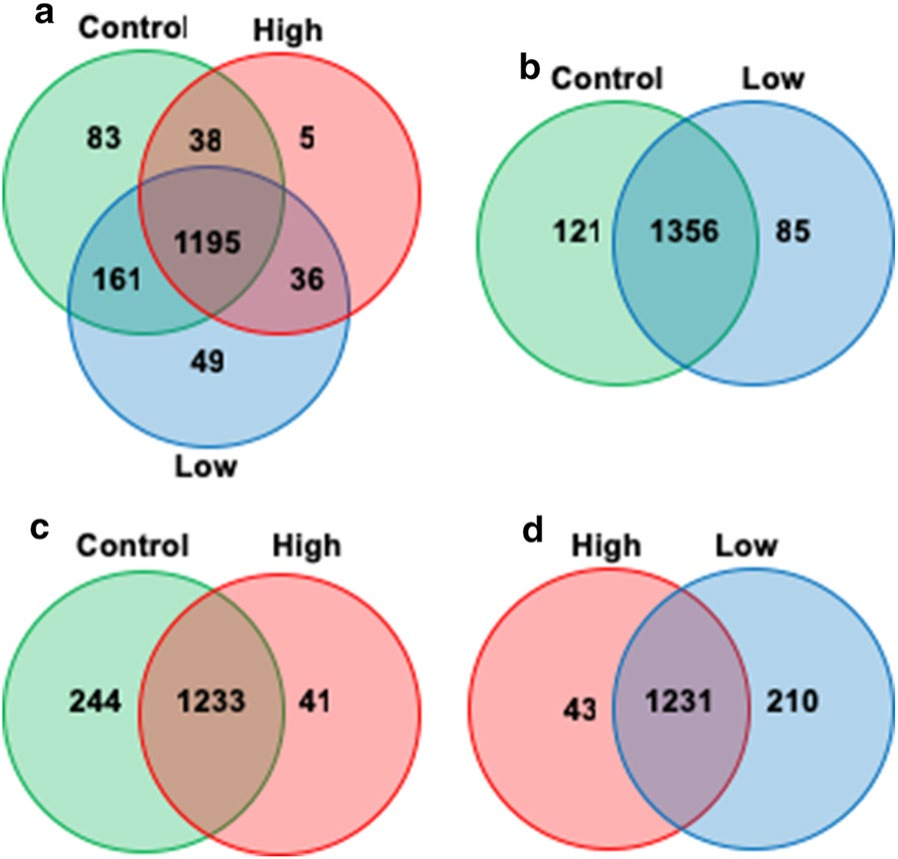
Protein identification comparisons between treatment groups in *An. gambiae* midguts. Midgut lysates from female *An. gambiae* mosquito midguts treated with varied concentrations of tBHP were subjected to a LC–MS/MS analysis to identify expressed proteins. **a** Proteins identified in all three experimental groups of control (untreated ex vivo organ culture media only), low (50 μM tBHP), and high (μM tBHP). **b** Proteins identified in control and low tBHP groups. **c** Proteins identified in control and high tBHP groups. **d** Proteins identified in high and low tBHP groups

We generated three experimental groups: (1) untreated ex vivo organ culture only (control), and two tBHP-treated groups exposed to (2) 50 μM (low), and (3) 200 μM (high). We identified a total of 1567 quantifiable proteins using the VectorBase *An. gambiae* protein FASTA sequence database (http://www.vectorbase.org, *Anopheles gambiae* PEST, AgamP4.2) [20] with MASCOT version 2.5 used as the search engine (Additional files 4, 5 and 6). The three experimental groups shared 1195 of the proteins (76.3%), while 83 proteins (5.3%) were found only in the control group, 49 proteins (3.13%) were found only in the low tBHP group, and 5 proteins (0.32%) were found only in the high tBHP group (Fig. 2a). Furthermore, proteomic profiles of the different experimental groups identified 1356 proteins (86.5.7%) shared between the control and low tBHP groups (Fig. 2b), 1233 proteins (78.6%) shared between control and high tBHP groups (Fig. 2c), and 1231 proteins (78.6%) shared between low and high tBHP groups (Fig. 2d).

We identified the antioxidant proteins, which fell into the following groups: heat shock proteins (HSP), cytochromes (CYT), Trx-dependent, and GSH-dependent proteins (Additional files 4, 5 and 6). However, only 20 antioxidant proteins out of total 1567 proteins (1.27%) were significantly enriched suggesting that the midgut cells were not initiating an antioxidant response (Additional files 4, 5 and 6). We hypothesized that they may be responding through other mechanisms to tBHP generated ROS.

**Fig. 3:**
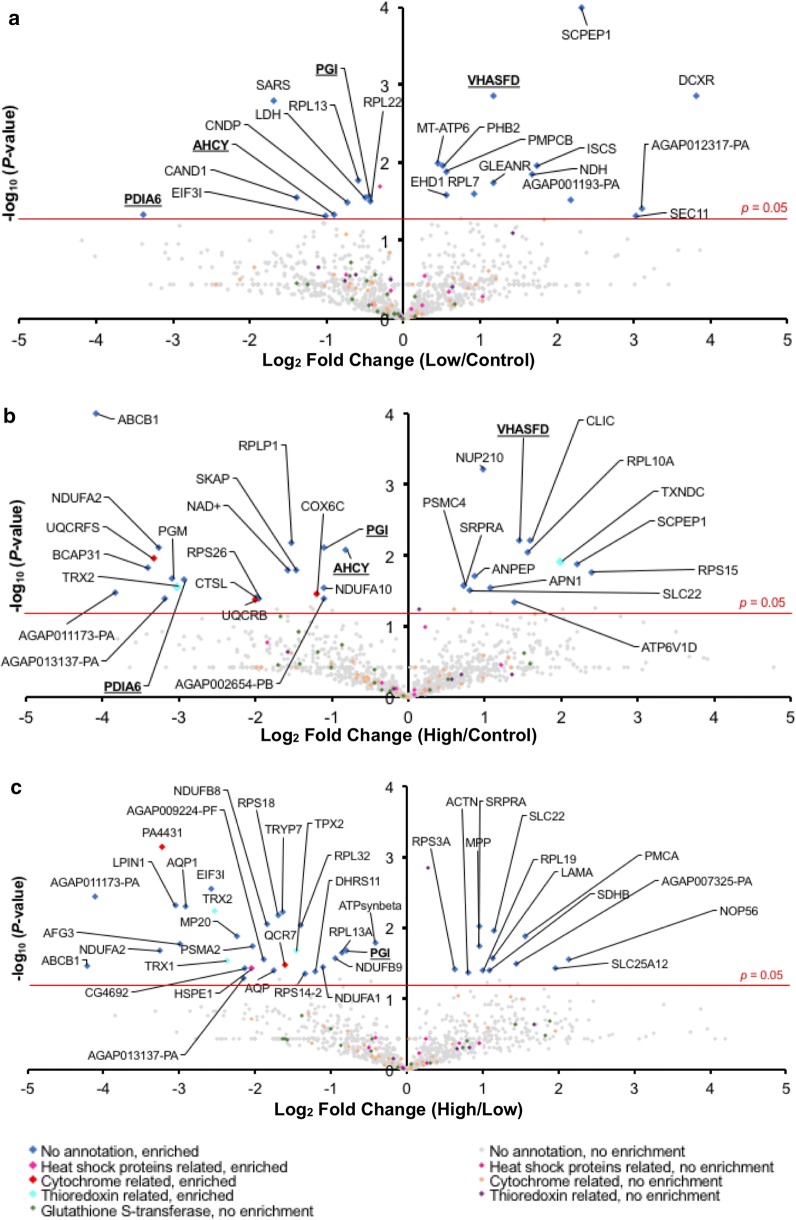
Female *An. gambiae* mosquito midguts treated with various concentrations of tBHP were subjected to a LC–MS/MS analysis to identify expressed proteins. Volcano plots of quantifiable protein comparisons. **a** Low (50 µM tBHP) versus control (untreated ex vivo organ culture only) experimental groups. **b** High (200 µM tBHP) versus control (untreated ex vivo organ culture only) experimental groups. **c** High (200 µM tBHP) versus low (50 µM tBHP) experimental groups. Significant fold change was calculated using Student’s t-test with *P*-value ≤ 0.05. Annotations of significantly enriched proteins are indicated

Further analysis of the proteomic profiles of the experimental groups based on their spectral count values (*P* ≤ 0.05; Student’s t-test; Fig. 3b–d; Additional files 4, 5 and 6) identified additional 89 proteins that were differentially expressed between the groups. Out of these, we found 10 proteins that were enriched (highly expressed) in the low treatment group (Fig. 3b and Additional file 4) and 18 proteins enriched in the high treatment group (Fig. 3c, d and Additional files 5, 6). Evaluation of the respective annotated functions of the enriched proteins revealed cellular roles in either ribosome biogenesis or in cellular trafficking as part of the lysosomal exocytosis machinery. In this report we will focus on proteins with annotated functions in ribosome biogenesis due to their close interlink to cellular stress response, including oxidative stress, and the potential application of the ribosome biogenesis machinery as a transmission blocking intervention for Malaria. Details about the other proteins involved in cellular trafficking and lysosomal exocytosis are shown in Additional file 7.

#### Alteration in ribosomal proteins (RPs) profile

Differential expression of several RPs was observed in *An. gambiae* midgut epithelial cells that were exposed to different treatments of tBHP (Fig. 3a–c; Table S1 of Additional file 8). In the low tBHP group we identified enrichment of 60S ribosomal protein L7 (RpL7) by 1.9-fold (Fig. 3a and Table S1 of Additional file 8). In this group, we also identified decreased expression of 60S L13 (RpL13) and L22 (RpL22) ribosomal proteins by 1.5- and 1.33-fold, respectively (Fig. 3a and Table S1 of Additional file 8).

In the high tBHP group we identified 60S ribosomal protein L10a (RPL10A), 40S ribosomal protein S15 (RPS15), 40S ribosomal protein S3a (RPS3A), 60S ribosomal protein L19 (RpL19), and a putative RNA binding protein enriched by 3.0-, 5.30-, 1.55-, 2.31-, and 2.72-fold, respectively (Fig. 3b, c and Table S1 of Additional file 8). In contrast, seven RPs showed reduced expression in the high treatment group: 60S ribosomal protein LP1 (RpLP1), 40S ribosomal protein S26 (RpS26), 60S ribosomal protein L32 (RpL32), 60S ribosomal protein L13a, (RpL13a), 60S ribosomal protein L11 (RpL11), 40S ribosomal protein S14 (RpS14), and 40S ribosomal protein S18 (RpS18) with 2.86-, 3.85-, 2.63-, 1.81-, 16.67, 2.5-, and 3.3-fold, respectively (Fig. 3b, c and Table S1 of Additional file 8). Taken together these results are an indication that challenge of mosquito epithelial cells with tBHP induces an altered expression of RPs.

### Discussion

*Anopheles gambiae* midgut epithelial cells are under frequent oxidative stress either from the digestion of ingestion blood meal or mosquito’s innate immunity against the invading *Plasmodium* parasite. In such cases, the epithelial cells need to have their antioxidant defenses highly expressed against the oxidative attack [15, 16]. We observed that the expression of *Ag*Trx-1, a key player in the cellular redox network, remains similar under different conditions of oxidative stress resulting from tBHP exposure. A plausible explanation for this is that the *Ag*Trx-1 baseline expression could already be high in midgut epithelial cells most likely due to its other cellular roles in addition to the antioxidant system, so no differential expression was observed [12, 13]. Considering this initial observation, we examined further the midgut proteomic profiles to identify oxidative stress proteins that are differentially expressed following tBHP treatment.

Examination of the midgut proteomic profile for redox-related proteins such as HSPs, CYTs, Trx-related, and GSH-related revealed a significantly small proportion of these proteins are enriched following tBHP treatment. This suggests that the midgut epithelial cells are responding to the oxidative stress following tBHP treatment through other non-redox related mechanisms.

We observed modified expression in several non-redox proteins, most notably an imbalance in the levels of RPs following treatment with tBHP. In an unstressed cell equimolar amounts of RPs are generated during ribosome biogenesis [21]. A change in the RP levels due to cellular stresses such as hypoxia, heat shock, ionizing radiation (IR), oxidative stress, and certain drugs could disrupt the balance and thus reduce the number of functional ribosomes impairing protein synthesis [22]. In response, the cell induces the ribosomal/nucleolar stress response to mitigate the loss in functional ribosomes [22]. In eukaryotic cells, the most common inducer of ribosomal stress response is the transactivation and accumulation of the tumour suppressor p53 caused by the inhibition of the E3 ubiquitin ligase activity of mouse double minute 2 (MDM2) homolog on p53 [23]. RPs can bind to the MDM2 homolog, inhibiting its E3 ubiquitin ligase activity on p53 which leads to activation of p53 [24]. However, certain invertebrates including the dipteran insect *Drosophila* lack a discernible MDM2 homolog [25]. Not surprisingly, *An. gambiae* also a dipteran, also lacks a discernible MDM2 homolog evident from a thorough BLAST search results of *An. gambiae* genome through the VectorBase (http://www.vectorbase.org, *An. gambiae* PEST, AgamP4.2) database (data not shown), which suggests that induction of ribosomal stress response uses an alternative mechanism [21, 26].

An imbalance in RP levels has been shown to be associated with the “Minute” phenotype in *Drosophila* [27]. The “Minute” phenotype is associated with increased expression of JNK signalling [28], which has been linked to a wide range of biological processes, including stress response and immunity [29, 30]. Interestingly, *Drosophila* homologs of the differentially expressed RPs in our *Anopheles* proteomic data have either been confirmed or predicted to be encoded by a “Minute” locus in the fruit fly [27]. We, therefore, postulate that the overall imbalance in the levels of RPs following tBHP treatment of *An. gambiae* midguts has the same consequence of increasing the expression of JNK signalling as seen in *Drosophila*. Increased expression in JNK signalling increases tolerance to oxidative stress in *Drosophila* as well as in *An. gambiae* [31]. Overexpression of the upstream member JNKK (Hemipterous; Hep) or down regulation of the downstream target *puckered* (puc) in *Drosophila* results in flies that exhibit an increased tolerance to oxidative stress [32]. Interestingly, in *An. gambiae,* JNK signalling regulates the gene oxidation resistance 1 (OXR1), which in turn regulates the expression of antioxidant enzymes such as Catalase and GPx [31].

Our data suggest that various inducers of ROS trigger a non-*Ag*Trx-1 pathway, that is likely dependent on the potency of the ROS-inducer. The *Ag*Trx-1 and ribosomal/nucleolar stress response may work in concert to maintain cellular/tissue homeostasis during blood feeding. The induction of ribosomal/nucleolar stress, as the additional response to oxidative stress, could be harnessed as a transmission-blocking strategy. A practical scenario could be the application of druggable small molecules that would induce high ROS activity in the mosquito blood meal bolus in the midgut during digestion (akin to levels induced by tBHP). This would create an environment of selective toxicity wherein the mosquito naturally survives due to its cooperative oxidative stress response pathways, but the parasite would be unable to manage the elevated oxidative stress, resulting in its arrested development and destruction by the mosquito and thus failure to be transmitted to the next human host.

## Limitations

A major limitation to this work is that measurement of ROS/RNS levels in both the control and treated midgut samples was not carried out due to the inherent technical difficulties with this system. This makes it difficult to ascertain if the response observed is entirely due to tBHP treatment. Furthermore, we were not able to carry out the transmission-blocking potential of tBHP in vivo against *Plasmodium* due to difficulty in getting the mosquitoes used in our assays to feed on a blood meal containing tBHP due to its mosquito repellency.

## Additional files

**Additional file 1.** Materials and Methods.

**Additional file 2.** Quantitative immunoblot data analysis.

**Additional file 3.** Protein identification and peptide information raw data.

**Additional file 4.** Proteomic data on differential expression between control and low treatment groups.

**Additional file 5.** Proteomic data on differential expression between control and high treatment groups.

**Additional file 6.** Proteomic data on differential expression between low and high treatment groups.

**Additional file 7.** Proteomic data on enriched (overexpressed) proteins with annotated functions in cellular trafficking and lysosomal exocytosis.

**Additional file 8.** Proteomic data on differentially expressed proteins with annotated functions in ribosomal/nucleolar stress (ribosomal biogenesis).

## Abbreviations

tBHP: tert-Butyl hydroperoxide
Trx: thioredoxin
GSH: glutathione
ROS: reactive oxygen species
RNS: reactive nitrogen species
SDS: sodium dodecyl sulphate
PAGE: polyacrylamide gel electrophoresis
LC: liquid chromatography
MS: mass spectrometer/spectrometry
ANOVA: analysis of variance
RP: ribosomal proteins
MDM2: mouse double minute 2
S: small subunit
L: large subunit
SCPEP1: serine carboxypeptidase 1
VHASFD: V-type transporting ATPase 54 kDa subunit
MT-ATP6: F-type H^+^ transporting ATPase
PMPCB: peptidase (mitochondrial processing) beta
GLEANR: female reproductive tract protease
EHD1: eps 15 homology domain-containing protein 1
SEC11: signal peptidase, ER-type
CLIC: chloride intracellular channel
ANPEP: alanyl aminopeptidase
SRPRA: signal recognition particle receptor alpha
PSMC4: 26S proteosome regulatory subunit T3
APN3: aminopeptidase N3
SLC22: solute carrier family 22
ATP6V1D: V-type H^+^ transporting ATPase subunit D
NUP210: nuclear pore complex protein glycoprotein 210
