# Supplementary material for: Ribosomal/nucleolar stress induction regulates tert-Butyl hydroperoxide (tBHP) mediated oxidative stress in Anopheles gambiae midguts
Source: BMC Res Notes. 2019 Mar 29;12:182. doi: 10.1186/s13104-019-4196-1 (PMC6440166; doi:10.1186/s13104-019-4196-1)
Supplement: Supplementary file 1 — Additional file 1. Materials and Methods. [file 13104_2019_4196_MOESM1_ESM.docx]

Additional file 1

Ribosomal/nucleolar stress induction regulates tert-butyl hydroperoxide (tBHP) mediated oxidative stress in *Anopheles gambiae* midguts

Brian B. **Tarimo**^1,2,3^, Bernadette A. **Hritzo**^2^, Henry Chun Hin **Law**^4,^*, Dingyin **Tao**^2,^*, Rebecca **Pastrana-Mena**^2^, Stefan M. **Kanzok**^5^, Joram J. **Buza**^1^, and Rhoel R. **Dinglasan**^1,2,4,†^

^†^Corresponding author: [rdinglasan@epi.ufl.edu](mailto:rdinglasan@epi.ufl.edu). Tel. +1-352-294-8448.

Emerging Pathogens Institute, 2055 Mowry Road, Rm 375, Gainesville, Florida 32611, USA.

***Ex-vivo* midgut organ culture media**

*Ex-vivo* studies were done using *An. gambiae* midguts maintained in a culture media that contained 3895 μL of RPMI 1640 without L-glutamine or phenol red (Quality Biologicals), 1 mL of heat-inactivated fetal bovine serum (Sigma-Aldrich), 100 μL of 10000U: 10mg/ml PenStrep (Cellgro) to a final concentration of 100U; 10 μg/mL, and 5 μL of 250 μg/ml amphotericin. Due to its sensitivity to light, Amphotericin was added to the media last and the final volume was immediately covered by foil to reduce light exposure. The media was equally split into 1.5 ml tubes each containing 1 ml and stored at -20^o^C until usage.

**Mosquito rearing, experimental treatments, and ROS induction assays**

*An. gambiae* (KEELE strain) mosquitoes were used for all the experimental treatments. These mosquitoes were maintained in an insectary at the Johns Hopkins Malaria Research Institute (JHMRI), at 26°C and 70% humidity with 12 hours light: dark cycles and supplemented with 10% sucrose solution.

ROS induction (oxidative stress) assays were performed using an *ex vivo* system. In these assays, 50 *An. gambiae* female mosquitoes (4-7 days old) were dissected and individual midguts collected in 1x PBS on ice. In each of the treatment groups the 50 midguts were split into two sub-groups: one containing five midguts (for SDS-PAGE and immunoblot analysis) and the other containing 45 midguts (for LC-MS/MS analysis). Midguts in both groups were submerged in 200 μL containing their respective concentrations of tBHP (Alfa Aesar, Haverhill, MA) in organ culture media and left for 15 minutes at room temperature away from light exposure due to sensitivity of amphotericin to light. Treatment media was then removed from the midgut samples followed by stringent wash of the samples with 1x PBS and storage at -20^o^C and -80^o^C until further SDS-PAGE/immunoblot and LC-MS/MS analyses, respectively. The treatments groups were: control (organ culture media only), 50 μM, 125 μM, 200 μM, 250 μM, 500 μM, and 1 mM. These experiments were replicated three times using independent biological cohorts of mosquitoes.

SDS-PAGE and Immunoblot analysis

Midgut lysates (5 midgut equivalents) of experimental groups were thawed on ice to RT and then heated at 95^o^C for 10 minutes. Approximately 15 μL of midgut lysates (~2.5 midguts per well) were loaded into a 4-20% Tris-glycine gel. Proteins were separated under reducing conditions at a constant 100V and then transferred to a nitrocellulose membrane. The membranes were blocked in a solution of Odyssey blocking buffer (Li-COR Biosciences, Lincoln, NE) then probed first with rabbit anti-*Ag*Trx-1 antiserum (obtained from S. Kanzok, Loyola University) (44 mg/ml) and second with rabbit anti-*An*APN-1 (RCB-A terminal) mAb used as a loading control diluted 1:200 and 1:1000 in a solution of Odyssey blocking buffer, respectively.

Mouse anti-rabbit IgG secondary antibodies labeled with IRDye 680RD (Li-COR Biosciences, Lincoln, NE) diluted 1: 20,000 in Odyssey blocking buffer was used to detect anti-*Ag*Trx-1 and anti-*An*APN-1 separately. Quantitative immunoblotting was performed by determining *Ag*Trx-1 expression levels relative to *An. gambiae* midgut *An*APN-1 (AGAP004809) using the Li-COR analytical software (version 3.0). All immunoblots were imaged using the Li-COR Odyssey infrared imaging system (Li-COR) and signal intensity was calculated in K counts mm^2^.

Extraction, solubilization, and digestion of proteins

Prior to LC-MS/MS analysis, treatment groups (45 midguts/samples) were processed for proteins. Total protein lysate was prepared by lysing the midgut samples with 45 μL of SDST-lysis buffer (4% SDS (w/v), 100 mM Tris/HCl, 0.1 M DTT pH 7.6) and boiled at 95^o^C for 5 minutes. 30 μL of the protein lysates was taken for protein digestion according to the Filter-Aided Sample Preparation (FASP) protocol previously described by Wiśniewski et al. ^1^ using a 10 kDa molecular weight cutoff filter (EMD Millipore, Billerica, MA) as previously described by Tao et al. ^2^ and Tweedell et al. ^3^. Acidified tryptic peptides from FASP approaches were desalted using an HPLC column and their concentrations determined by BCA as previously described Tao et al. ^2^ and Tweedell et al. ^3^.

Online 2D LC-MS/MS analysis

Peptide products desalted and digested by the FASP protocol were dissolved in loading buffer (97.9% water, 2% ACN, and 0.1% formic acid (FA)) and ~ 20 μg was injected to our previously constructed online 2D HPLC-MS/MS system, using the exact method as described previously by Tao et al. ^2^, Tao et al. ^4^, and Tweedell et al ^3^.

Database searching and label-free quantification analysis

All the LC-MS/MS raw data were converted to Mascot generic format (.mgf) by Agilent MassHunter Qualitative Analysis B.04.00. The data acquired was used to search the VectorBase *Anopheles gambiae* protein FASTA sequence database (VectorBase, http://www.vectorbase.org, *Anopheles gambiae* PEST, AgamP4.2.) for peptide sequence alignments. The search engine used for the search was MASCOT version 2.5 with the following parameters: precursor ion mass tolerance of 50 ppm, fragment ion mass tolerance of 0.2 Da, carbamidomethylation of cysteine and oxidation of methionine residues set as fixed and variable modifications respectively. Peptides were searched using fully tryptic cleavage constraints, and up to two internal cleavage sites were allowed for tryptic digestion.

The MASCOT search results were exported as .DAT format and then imported into the Scaffold software (version 4.4.5, Proteome Software) for curation, label-free quantification, analysis, and visualization. Overall, protein false discovery rates of less than 1% and peptide false discovery rates of less than 1% were obtained with Scaffold filters, and each protein had ≥ 2 unique peptides. Identified proteins were clustered to remove redundancy. Proteins were clustered together if there was a peptide identification shared between them, because this indicates substantial sequence similarity, and the protein with the greatest number of peptides identified was considered the unique protein identification from that group. The data analysis pipeline meets all MIAPE standards ^5^ and the detailed peptide data can be found in Additional file 3.

Statistical analyses

For quantitative immunoblot analyses, relative expression of *Ag*Trx-1 was calculated using a one-way multi-variable analysis of variance (ANOVA). Proteins quantification to identify enriched proteins between treatment groups was carried out by Student’s t-test. All statistical analyses were carried out using the software GraphPad Prism (version 6.0e). The *P-*values of ≤0.05 were considered statistically significant. All experimental reactions were carried using at least three independent replicate samples.

**References**

(1) Wiśniewski, J. R.; Zougman, A.; Nagaraj, N.; Mann, M. Universal Sample Preparation Method for Proteome Analysis. *Nat. Methods* **2009**, *6* (5), 359–362.

(2) Tao, D.; King, J. G.; Tweedell, R. E.; Jost, P. J.; Boddey, J. a.; Dinglasan, R. R. The Acute Transcriptomic and Proteomic Response of HC-04 Hepatoma Cells to Hepatocyte Growth Factor and Its Implications for *Plasmodium Falciparum* Sporozoite Invasion. *Mol. Cell. Proteomics* **2014**, *13* (5), 1153–1164.

(3) Tweedell, R.; Tao, D.; Dinglasan, R. R. The Cellular and Proteomic Response of Primary and Immortalized Murine Kupffer Cells Following Immune Stimulation Diverges from That of Monocyte-Derived Macrophages. *Proteomics* **2015**, *15* (2–3), 545–553.

(4) Tao, D.; Ubaida-Mohien, C.; Mathias, D. K.; King, J. G.; Pastrana-Mena, R.; Tripathi, A.; Goldowitz, I.; Graham, D. R.; Moss, E.; Marti, M.; et al. Sex-Partitioning of the *Plasmodium Falciparum* Stage V Gametocyte Proteome Provides Insight into Falciparum-Specific Cell Biology. *Mol. Cell. Proteomics* **2014**, *13* (10), 2705–2724.

(5) Taylor, C. F.; Paton, N. W.; Lilley, K. S.; Binz, P.-A.; Jr, R. K. J.; Jones, A. R. The Minimum Information about a Proteomics Experiment (MIAPE). *Nat. Biotechnol.* **2007**, *25* (8), 887–893.
