# Supplementary material for: Ribosomal/nucleolar stress induction regulates tert-Butyl hydroperoxide (tBHP) mediated oxidative stress in Anopheles gambiae midguts
Source: BMC Res Notes. 2019 Mar 29;12:182. doi: 10.1186/s13104-019-4196-1 (PMC6440166; doi:10.1186/s13104-019-4196-1)
Supplement: Supplementary file 7 — Additional file 7. Proteomic data on enriched (overexpressed) proteins with annotated functions in cellular trafficking and lysosomal exocytosis. [file 13104_2019_4196_MOESM7_ESM.docx]

**Additional File 7**

**Table S1.** Proteins enriched with function in either posttranslational modification, cellular transport, or energy metabolism.

| **Protein description** | **tBHP**  **Group** | **Fold**  **change** | ***P-*value** | **Function** | **Signal**  **peptide (Y/N)** |
| --- | --- | --- | --- | --- | --- |
| **SCPEP1** (AGAP011442)  Serine carboxypeptidase 1 | Low | 5.00 | 0.0001 | Posttranslational processing ^1^ | Y |
| **VHASFD**(AGAP009486)  V-type transporting ATPase 54 kDa subunit | Low | 2.30 | 0.0014 | Electrogenic pump ^2^ | N |
| **MT-ATP6** (AGAP005134)  F-type H^+^ transporting ATPase | Low | 1.40 | 0.01 | Energy metabolism ^3^ | Y (mTP) |
| **ISCS** (AGAP009094)  cysteine desulfurase | Low | 3.30 | 0.011 | Biosynthesis of iron-sulfur (Fe-S) clusters^4^ | Y (mTP) |
| **PMPCB** (AGAP005558)  peptidase (mitochondrial processing) beta | Low | 1.5 | 0.013 | Catalyses the cleavage of nascent/pre-proteins newly imported into the mitochondria ^5^ | Y (mTP) |
| **NDUFV1** (AGAP010039)  NADH dehydrogenase [ubiquinone] flavoprotein1, mitochondrial | Low | 3.20 | 0.014 | Energy metabolism ^6^ | Y (mTP) |
| **GLEANR** (AGAP008861)  Female reproductive tract protease | Low | 2.2 | 0.018 | Posttranslational processing | Y |
| **EHD1** (AGAP004593)  Eps 15 homology domain-containing protein 1 | Low | 1.5 | 0.026 | Cellular transport of compounds ^7^ | N |
| **SEC11** (AGAP003069)  Signal peptidase, ER-type | Low | 8.2 | 0.048 | Posttranslational processing^1^ | N |
| **CLIC** (AGAP000943)  Chloride intracellular channel | High | 3.1 | 0.0061 | Chloride ion transport across membranes ^8^ | N |
| **VHASFD** (AGAP009486)  V-type transporting ATPase 54 kDa subunit | High | 2.80 | 0.0061 | Electrogenic pump ^9^ | N |
| **SCPEP1** (AGAP011442)  Serine carboxypeptidase 1 | High | 4.70 | 0.013 | Posttranslational processing ^1^ | Y |
| **ANPEP** (AGAP012745)  Alanyl aminopeptidase | High | 1.9 | 0.019 | Peptidase activity; posttranslational modification ^10,11^ | N |
| **SRPRA** (AGAP010894)  Signal recognition particle receptor alpha | High | 1.7 | 0.026 | Cellular transport ^12,13^ | Y |
| **PSMC4** (AGAP003008)  26Sproteosome regulatory subunit T3 | High | 1.7 | 0026 | Protein homeostasis ^14^ | N |
| **APN3** (AGAP013255)  Aminopeptidase N3 | High | 2.1 | 0.028 | Peptidase activity; posttranslational modification ^15^ | Y |
| **SLC22** (AGAP004309)  Solute carrier family 22 | High | 1.8 | 0.031 | Cellular transport ^16^ | N |
| **ATP6V1D**(AGAP010298)  V-type H^+^ transporting ATPase subunit D | High | 2.6 | 0.045 | Electrogenic pump ^17^ | Y (mTP) |
| **NUP210** (AGAP006280)  Nuclear pore complex protein glycoprotein 210 | High | 2.0 | 0.0006 | Cellular transport | N |

In the first column, the name of the protein is described with its abbreviated form and accession number. The second column indicates the treatment group. The third column shows the fold change in enrichment level for each of the described proteins. The fourth column shows the *P*-value (*P*≤0.05) results of Student’s t-test comparisons on the fold change in enrichment level. Only P-values that are significant are provided. The fifth column describes the function of the described protein and associated references and the last column denotes whether the protein contains a signal peptide. Y = yes, N = no, and mTP = mitochondrial Target Peptide.

**Reference**

(1) Pshezhetsky, A. V.; Hinek, A. Serine Carboxypeptidases in Regulation of Vasoconstriction and Elastogenesis. *Trends in Cardiovascular Medicine*. 2009, pp 11–17.

(2) Forgac, M. Vacuolar ATPases: Rotary Proton Pumps in Physiology and Pathophysiology. *Nature Reviews Molecular Cell Biology*. 2007, pp 917–929.

(3) Yoshida, M.; Muneyuki, E.; Hisabori, T. ATP Synthase--a Marvellous Rotary Engine of the Cell. *Nat. Rev. Mol. Cell Biol.* **2001**, *2* (9), 669–677.

(4) Braymer, J. J.; Lill, R. Iron–Sulfur Cluster Biogenesis and Trafficking in Mitochondria. *Journal of Biological Chemistry*. 2017, pp 12754–12763.

(5) Mossmann, D.; Meisinger, C.; Vögtle, F. N. Processing of Mitochondrial Presequences. *Biochimica et Biophysica Acta - Gene Regulatory Mechanisms*. 2012, pp 1098–1106.

(6) Yagi, T.; Matsuno-Yagi, A. The Proton-Translocating NADH-Quinone Oxidoreductase in the Respiratory Chain: The Secret Unlocked. *Biochemistry*. 2003, pp 2266–2274.

(7) Kieken, F.; Jović, M.; Naslavsky, N.; Caplan, S.; Sorgen, P. L. EH Domain of EHD1. *J. Biomol. NMR* **2007**, *39* (4), 323–329.

(8) Littler, D. R.; Harrop, S. J.; Goodchild, S. C.; Phang, J. M.; Mynott, A. V.; Jiang, L.; Valenzuela, S. M.; Mazzanti, M.; Brown, L. J.; Breit, S. N.; et al. The Enigma of the CLIC Proteins: Ion Channels, Redox Proteins, Enzymes, Scaffolding Proteins? *FEBS Letters*. 2010, pp 2093–2101.

(9) Nishi, T.; Forgac, M. The Vacuolar (H+)-ATPases--Nature’s Most Versatile Proton Pumps. *Nat. Rev. Mol. Cell Biol.* **2002**, *3* (2), 94–103.

(10) Zhang, X.; Xu, W. Aminopeptidase N (APN/CD13) as a Target for Anti-Cancer Agent Design. *Curr. Med. Chem.* **2008**, *15* (27), 2850–2865.

(11) Bauvois, B.; Dauzonne, D. Aminopeptidase-N/CD13 (EC 3.4.11.2) Inhibitors: Chemistry, Biological Evaluations, and Therapeutic Prospects. *Med. Res. Rev.* **2006**, *26* (1), 88–130.

(12) Gilmore, R.; Walter, P.; Blobel, G. Protein Translocation across the Endoplasmic Reticulum. II. Isolation and Characterization of the Signal Recognition Particle Receptor. *J. Cell Biol.* **1982**, *95* (2 Pt 1), 470–477.

(13) Gilmore, R.; Blobel, G.; Walter, P. Protein Translocation across the Endoplasmic Reticulum. I. Detection in the Microsomal Membrane of a Receptor for the Signal Recognition Particle. *J. Cell Biol.* **1982**, *95* (3), 463–469.

(14) Hiller, M. M.; Finger, A.; Schweiger, M.; Wolf, D. H. ER Degradation of a Misfolded Luminal Protein by the Cytosolic Ubiquitin-Proteasome Pathway. *Science* **1996**, *273* (5282), 1725–1728.

(15) Luan, Y.; Xu, W. The Structure and Main Functions of Aminopeptidase N. *Curr. Med. Chem.* **2007**, *14* (6), 639–647.

(16) Koepsell, H. The SLC22 Family with Transporters of Organic Cations, Anions and Zwitterions. *Molecular Aspects of Medicine*. 2013, pp 413–435.

(17) Dow, J. A. T. The Multifunctional *Drosophila Melanogaster* V-ATPase Is Encoded by a Multigene Family. *J. Bioenerg. Biomembr.* **1999**, *31* (1), 75–83.
