# Supplementary material for: Ribosomal/nucleolar stress induction regulates tert-Butyl hydroperoxide (tBHP) mediated oxidative stress in Anopheles gambiae midguts
Source: BMC Res Notes. 2019 Mar 29;12:182. doi: 10.1186/s13104-019-4196-1 (PMC6440166; doi:10.1186/s13104-019-4196-1)
Supplement: Supplementary file 8 — Additional file 8. Proteomic data on differentially expressed proteins with annotated functions in ribosomal/nucleolar stress (ribosomal biogenesis). [file 13104_2019_4196_MOESM8_ESM.docx]

**Additional File 8**

**Table S1.** Ribosomal protein abundance following treatment with low and high tBHP concentrations. Gray highlighted boxes indicate overexpressed proteins and unshaded represent down-regulated expression.

| **Protein description** | **tBHP Group** | **Fold**  **change** | **P-value** | **Reference** |
| --- | --- | --- | --- | --- |
| **RpL7** (AGAP008916) 60S ribosomal protein L7 | Low | 1.9 | 0.025 | Protein translation machinery ^1^ |
| **RpL10A** (AGAP011298)  60S ribosomal protein L10a | High | 3.0 | 0.0088 | Protein translation machinery ^2^ |
| **RpS3A** (AGAP003532)  40S ribosomal protein S3a | High | 1.50 | 0.038 | Protein translation machinery^3^ |
| **RpL19** (AGAP004422)  60S ribosomal protein L19 | High | 2.0 | 0.04 | Protein translation machinery^4^ |
| **AGAP007325**  Putative RNA binding protein. | High | 2.7 | 0.032 | None |
| **RpL13** (AGAP001805)  60S ribosomal protein L13 | Low | 1.50 | 0.017 | Protein translation machinery^3^ |
| **RpL22 (**AGAP005046**)**  60S ribosomal protein L22 | Low | 1.33 | 0.031 | Protein translation machinery^4^ |
| **RpLP1** (AGAP007740)  60S ribosomal protein LP1 | High | 2.86 | 0.0067 | Protein translation machinery^4^ |
| **RpS26** (AGAP012100)  40S ribosomal protein S26 | High | 3.85 | 0.041 | Protein translation machinery ^4^ |
| **RpL32** (AGAP002122)  60S ribosomal protein L32 | High | 2.9 | 0.0092 | Protein translation machinery ^4^ |
| **RpL13A** (AGAP010257)  60S ribosomal protein L13a | High | 1.7 | 0.022 | Protein translation machinery ^4^. |
| **RpL11** (AGAP011173)  60S ribosomal protein L11 | High | 16.67 | 0.0036 | Protein translation machinery ^1^ |
| **RpS14** (AGAP002346)  40s ribosomal protein S14 | High | 2.5 | 0.044 | Protein translation machinery ^4^ |
| **RpS18** (AGAP028693)  40S ribosomal protein S18 | High | 3.3 | 0.0065 | Protein translation machinery ^4^ |

In the first column, the protein is described in its abbreviated form. In the second column, the treatment group where the protein was enriched is mentioned. The third group shows the fold change in enrichment level for each of the described protein. In the fourth column, P-value (P≤0.05) results of Student’s t-test on the fold change in enrichment level are reported for each of the described protein. Only P-values that are significant are given.

**References:**

(1) Fromont-Racine, M.; Senger, B.; Saveanu, C.; Fasiolo, F. Ribosome Assembly in Eukaryotes. *Gene*. 2003, pp 17–42.

(2) Koga, M.; Shichijo, S.; Yamada, A.; Ashihara, J.; Sawamizu, H.; Kusukawa, J.; Itoh, K. Identification of Ribosomal Proteins S2 and L10a as Tumor Antigens Recognized by HLA-A26-Restricted CTL. *Tissue Antigens* **2003**, *61* (2), 136–145.

(3) Wang; Pakpour, N.; Napoli, E.; Drexler, A.; Glennon, E. K. K.; Surachetpong, W.; Cheung, K.; Aguirre, A.; Klyver, J. M.; Lewis, E. E.; et al. *Anopheles Stephensi* P38 MAPK Signaling Regulates Innate Immunity and Bioenergetics during *Plasmodium Falciparum* Infection. *Parasit. Vectors* **2015**, *8* (1), 424.

(4) Marygold, S. J.; Roote, J.; Reuter, G.; Lambertsson, A.; Ashburner, M.; Millburn, G. H.; Harrison, P. M.; Yu, Z.; Kenmochi, N.; Kaufman, T. C.; et al. The Ribosomal Protein Genes and Minute Loci of *Drosophila Melanogaster*. *Genome Biol.* **2007**, *8* (10).
